# Graph neural network-based mutation-aware regression test ordering using code dependency graphs and execution traces^☆^

**DOI:** 10.1016/j.mex.2025.103782

**Published:** 2025-12-25

**Authors:** S Sowmyadevi, Anna Alphy

**Affiliations:** Department of Computer Science and Engineering, SRM Institute of Science and Technology, Delhi NCR campus, Ghaziabad, Utter Pradesh 201204, India

**Keywords:** Mutation testing, Test prioritization, Graph neural networks, Regression testing, APFD, Fault Detection, Software quality assurance

## Abstract

The mutation-aware test prioritisation system in this paper uses Graph Neural Networks (GNNs) to combine static program structure, dynamic execution traces, and mutation coverage into a hybrid graph representation to enhance regression testing. The framework embeds higher-order dependencies in test cases using GCN, GAT, and GraphSAGE variations and ranks them using a multi-objective optimisation function that balances fault detection, execution cost, and mutation coverage. On benchmark datasets like Defects4J and ManySStuBs4J, the proposed approach consistently outperforms traditional baselines (coverage-based APFD = 72.4 %, cost-based = 74.5 %) and ML baselines (LSTM = 80.1 %, RL = 82.7 %), achieving an average APFD of 88.9 % and mutation score of 84.6 % with a 16.1-second execution overhead. Statistical tests (Wilcoxon signed-rank, *p* < 0.05) indicate the robustness of these gains. Ablation experiments show that removing execution traces or mutation characteristics reduces APFD by 5–8 %, emphasising their relevance. Qualitative research shows that GNN embeddings cluster fault-related test cases for interpretable prioritisation. The suggested paradigm for contemporary regression testing is scalable, accurate, and mutation-driven.

• **Multi-Tiered Graph-Based Architecture**: The method transforms raw program artifacts (codebase, mutants, test traces) into Program Dependence Graphs and Call Graphs, where nodes represent program elements and edges capture dependencies enriched with runtime characteristics.
• **GNN-Powered Multi-Objective Optimization**: Core innovation uses Graph Neural Networks (GCN, GAT, GraphSAGE) to create enriched embeddings through iterative neighborhood aggregation, feeding into a scoring function that balances fault detection potential, execution cost, and mutation coverage.
• **Superior Validated Performance**: Achieves 88.9 % APFD compared to 82.7 % for best baseline methods on real-world datasets, with statistical significance confirmed through Wilcoxon signed-rank tests across multiple evaluation metrics.

## Graphical abstract

**Figure:**
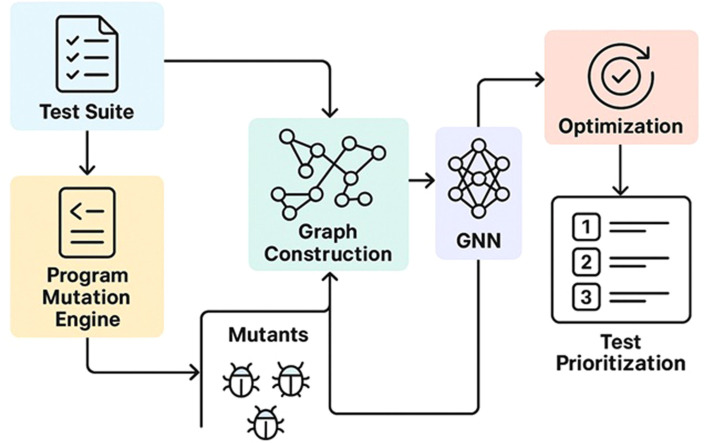

## Specifications table

**Table:** 

**Subject area**	Computer Science
**More specific subject area**	Software Engineering and Testing / Machine Learning for Software Quality Assurance
**Name of your method**	Mutation-Aware Graph Neural Network-based Test Case Prioritization
**Name and reference of original method**	Li, Y., Dang, X., Pian, W., Habib, A., Klein, J., & Bissyandé, T. F. (2024). Test input prioritization for graph neural networks. *IEEE Transactions on Software Engineering, 50*(6), 1396–1424. Chilese, M., Mitev, R., Orenbach, M., Thorburn, R., Atamli, A., & Sadeghi, A. R. (2024, May). One for all and all for one: Gnn-based control-flow attestation for embedded devices. In *2024 IEEE Symposium on Security and Privacy (SP)* (pp. 3346–3364). IEEE.
**Resource availability**	Real-world datasets from Defects4J, ManySStuBs4J, and problems.jar repositories; Mutation testing tools (PIT for Java, Major, MuJava); Graph construction from Program Dependence Graphs (PDGs) and Call Graphs

## Background

Regression testing is essential to modern software development, especially as applications become more sophisticated and expand quickly. Coverage-based algorithms and historical failure patterns commonly used to organise regression tests struggle to find faults efficiently. This gap has spurred interest in using sophisticated representation learning methods, such as Graph Neural Networks (GNNs), to improve test prioritisation through structural and behavioural insights [1]. Test cases that kill the mutant are prioritised because they are more sensitive to flaws and hence more important for efficient detection [2]. By embedding mutation-awareness within a GNN architecture, the system learns test case embeddings that capture structural location in the code dependency graph and behavioural sensitivity from execution traces. After ranking and arranging regression test cases using these embeddings, early defect identification improves significantly above coverage-driven or other machine learning baselines [3]. This method's relevance may be understood by considering adjacent fields' foundations and current advancement. GraphPrior showed mutation-based ranking algorithms that discover fault-revealing inputs using GNN-oriented designs for deep neural network test prioritisation [4]. GraphPrior is designed for neural network models, but its success shows the potential of mutation analysis and graph learning. While code-dependency graphs like Code Property Graphs can capture syntactic, control-flow, and data dependencies in a single graph structure, they are popular for vulnerability discovery and clone identification [5]. GNNs applied to execution traces can uncover silent buffer overflow vulnerabilities via data-flow graph augmentation [6]. The suggested regression test ordering approach integrates structural representation, behavioural context, and mutation sensitivity to provide a comprehensive and powerful representation.

Dang et al. found that regression testing prioritises test cases to promote early issue identification by arranging tests to maximise effectiveness [7]. Coverage-, cost-, and history-based techniques are classic. A series of empirical experiments by Shin et al. examined prioritisation methods utilising statement coverage and fault exposure probability [8]. In a review of test case prioritisation methods, She et al. found that reinforcement learning-based methods, such as RL-based Hidden Markov Models in GUI regression testing, outperformed pure statement coverage (∼0.61) in average fault detection (APFD ∼0.68) [9]. Another comprehensive review assessed greedy, meta-heuristic, and data-mining heuristics for coverage and fault detection utilising APFD, APBC, and APDC [10]. Antal et al. found that mutation testing, while effective for finding weak places with created mutants, is computationally costly with large numbers of mutants [11]. Mutation analysis can improve prioritisation but at a high runtime cost, and many methods miss deeper code/test relationships [12]. Wu developed DeepOrder, a regression-based deep learning model that ranks test cases in continuous integration contexts using historical execution data like duration and outcome. DeepOrder outperformed industry baselines and earlier approaches in defect identification and time efficiency [13]. NNE-TCP, introduced by Mitra et al., employs neural-network embeddings to map modified files to test cases by mapping them into multidimensional vectors and grouping by similarity, prioritising tests associated to recent changes and avoiding redundant executions [14]. These supervised approaches—regression models, clustering, and embeddings—improve performance, but they focus on local artefacts (e.g., test history or file linkage) and fail to model structural dependencies across code and test suite.

Software engineering activities like code representation, fault localisation, and vulnerability identification are increasingly using graph neural networks (GNNs) to train over structured representations like ASTs, CFGs, or Program Dependency Graphs. Chilese et al. compared graph convolutional networks (GCNs) and graph attention networks (GATs) to random, coverage-based, and history-based algorithms for automated test case prioritisation. Research found that GNN-based approaches, particularly GCNs, yielded superior APFD scores (≈84.2 %) despite obstacles such as computational complexity, scalability issues, data availability, and interpretability [15]. Sheikh et al. and Vélez et al.'s foundational surveys have shown GNNs can simultaneously learn structural and semantic properties from program graphs [16,17]. Code property graphs (CPGs)—which represent syntactic, control-flow, and data-dependency features of code—have been employed for vulnerability research and security, suggesting they may be used for test case modelling [18].

## Method details

### Higher-Level architecture for mutation-aware test prioritisation

The proposed mutation-aware test prioritisation architecture is tiered from raw program artefacts to actionable prioritisation recommendations led by graph learning and optimisation. The input layer collects three key data sources initially. First, it examines codebase usually big, real-world software projects that underpin test execution. Secondly, it combines mutants from mutation testing tools like PIT for Java. Mutants represent purposefully seeded software errors as proxy for true problems. Third, it records test case execution traces, including path coverage, execution order, and performance metrics. These inputs form the empirical basis for prioritisation decisions.

The program's static and dynamic interactions are formalised as graphs in the following step. PDGs or Call Graphs are created with nodes representing program elements like methods, statements, or functions and edges representing data or control dependencies like variable flow or branching conditions. Execution traces as node or edge characteristics capture path coverage frequency and execution duration to supplement these graphs with runtime information. This hybrid model lets the machine reason about dynamic behavioural signals and static program structure. The graph may be represented mathematically as G=(V,E,X), where V signifies nodes,E edges, and X is the feature matrix produced from execution traces and mutation data.

The system's core, the Graph Neural Network (GNN) module, compactly embeds test case, mutant, and program structural connections. GCN, GAT, and GraphSAGE models encapsulate higher-order graph dependencies. Iterative neighbourhood aggregation yields node embeddings $h_{v}^{k}$ are updated as:

(1) $$h_{v}^{\left(k\right)}=\sigma \left(\sum_{u\in N\left(v\right)}\frac{1}{cvu}W^{\left(k\right)h_{u}^{\left(k-1\right)}}\right),$$

where N(v) denotes the neighbors of node v, $c_{\left(vu\right)}$ is a normalization constant, $W^{k}$ the learnable weight matrix at layerk, and σa non-linear activation function. In this way, test case embeddings become enriched with both code-level semantics and the contextual information arising from mutants.

Based on these representations, the prioritisation engine ranks test cases using multi-objective optimisation. Balance fault detection, execution cost, and mutation coverage. A score function $S\;(t_{i})$ expressed as:

(2) $$S\left(t_{i}\right)=\alpha \cdot FD\left(t_{i}\right)+\beta \cdot \frac{1}{C\left(t_{i}\right)}+\gamma \cdot MC\left(t_{i}\right),$$

where $FD(t_{i})$ measures the fault detection potential of test case $t_{i}$ is its execution cost, and $MC(t_{i})$ denotes the proportion of mutants covered. The parameters α,β,γ are tunable weights that enable trade-offs depending on project-specific requirements. By applying this scoring mechanism, the system produces an ordered sequence of test cases that maximizes early fault revelation while minimizing redundant or costly executions.

Finally, the assessment layer runs the reordered test suite and quantifies KPI improvements. Prioritisation efficacy is measured by APFD, execution cost reduction, mutation score improvements, and defect discovery rate. APFD is formalised as:

(3) $$APFD=1-\frac{\sum_{i=1}^{m}Ti}{nm}+\frac{1}{2n},$$

where m represents the total number of faults,n the number of test cases, and $T_{i}$ the position of the first test case detecting fault i. Higher APFD values signify better prioritization efficiency. Through these evaluations, the system validates whether the integration of GNN-based representations and mutation-aware scoring mechanisms leads to measurable gains in testing effectiveness.

This study relies on a strong dataset from real-world repositories like Defects4J, ManySStuBs4J, and problems.jar, which comprise a wide range of Java applications with documented problems and test suites. These repositories include broken and corrected software versions to ensure experiment reliability and repeatability. Mutation testing using PIT, Major, or MuJava generates mutants by inserting false defects into the code to enhance the dataset. The suggested system's fault detection is tested using these mutations to mimic probable flaws. Run test suites on genuine faults and injected mutants to capture detailed execution traces after mutation production. These traces contain runtime data such coverage patterns, code fragment execution frequency, and success or failure indications, which are used for graph-based analysis.

## Construction of graphs

After dataset preparation, source code is turned into structured graphs. Programme Dependence Graphs (PDGs) and call graphs represent structural and semantic interactions between programme components. Each graph node represents a statement, method, or block, while edges represent control, data, and function call dependencies. Nodes are tagged with execution trace attributes to add runtime knowledge to graphs. Nodes store coverage frequency, execution cost, and the mutation-kill ratio, which shows how many mutants test cases killed. Formally, if $M_{c}$ represents the number of mutants killed by a test case and $M_{t}$ represents the total mutations it covers, the mutation-kill ratio is:

(4) $$R_{\left(mk\right)}=\frac{M_{c}}{M_{t}}$$

This enhanced graph shows static code structure and dynamic runtime behaviour.

### Features engineering

Feature engineering is crucial to graph representation. Statement type (assignment, branch, or method call), line coverage by test cases, and mutation coverage are node-level semantic and execution properties. Edge characteristics measure dependencies, such as control-flow edges weighted by branch execution frequency and data-flow edges by common variable count. Runtime behaviour can be reflected by weighting call relation edges by invocation numbers. Global graph properties capture project aspects beyond local features. A project's historical defect detection rate can indicate test suite efficacy, as can its size measures like classes, methods, and lines of code. By merging node, edge, and global characteristics, the framework captures fault detection dynamics in detail.

### Graph neural network design

After defining the graph format and feature set, create a fault-detecting Graph Neural Network (GNN). GCN, GAT, or GraphSAGE can be used depending on dependency complexity. These models convey feature information via graph neighbourhoods, letting nodes accumulate contextual signals from dependencies.

(5) $$P\left(T_{i}\right)\approx P_{r}\left(fault\;detected\;by\;T_{i}\right)$$

The loss function has two parts:

Encouragement to maximise mutant discovery through mutation coverage loss.

Minimising fault detection prediction error between expected and actual results.

Joint loss is formalised as:

(6) $$L=\alpha \cdot L_{mutation}+\left(1-\alpha \right)\cdot L_{fault}$$

where α is a tunable parameter balancing the two objectives. This ensures that the GNN does not overfit to artificial faults while maintaining accuracy for real-world bug detection.

### Optimising priorities

Test case execution order is optimised using GNN predictions. This stage prioritises tests likely to find errors early by ranking raw probability. Learning-to-rank approaches directly optimise ordering based on labelled outcomes, whereas Pareto-based multi-objective optimisation balances fault detection rate, execution cost, and redundancy. The prioritised sequence frontloads high-impact test cases to save testing resources. Mutation aware GNN based test case prioritisation is explained as in Algorithm 1.

**Algorithm 1:** Mutation-aware GNN-based test case prioritisation.

**Input:**
Dataset repositories D∈{Defects4J,ManySStuBs4J,problems.jar}
Mutation tools M∈{PIT,Major,MuJava}
Test suite $T=\{T_{1},T_{2},\ldots ,T_{n}\}$
**Output:**
Prioritised test sequence $T^{*}$
**Step 1: Dataset Preparation**
1.1 Extract faulty and corrected program versions from repositories D.
1.2 For each program version, execute mutation tools M to generate artificial mutants.
1.3 Execute test cases $T_{i}\in \;T$ against real faults and mutants.
1.4 Record execution traces: coverage patterns, frequency of execution, and pass/fail status.
**Step 2: Graph Construction**
2.1 Build Program Dependence Graphs (PDG) and Call Graphs.
2.2 Define nodes as program statements, methods, or blocks.
2.3 Define edges as control-flow, data-flow, or call dependencies.
2.4 Annotate nodes with attributes:
• Coverage frequency
• Execution cost
• Mutation-kill ratio
$R_{mk}=\;\frac{M_{c}}{M_{t}}$
where $M_{c}\;$is mutants killed by test case, $M_{t}\;is\;$mutants covered.
**Step 3: Feature Engineering**
3.1 Encode node features: statement type, line coverage, mutation coverage.
3.2 Encode edge weights:
• Control-flow → branch execution frequency
• Data-flow → shared variable count
• Call edges → invocation frequency
3.3 Add global graph properties: defect detection history, project size (LOC, classes, methods).
**Step 4: Graph Neural Network (GNN) Model**
4.1 Select model variant GNN∈{GCN,GAT,GraphSAGE}
4.2 Train GNN to predict probability of fault detection for each test case:
$P\left(T_{i}\right)\approx P_{r}\left(fault\;detected\;by\;T_{i}\right)$
4.3 Define joint loss:
$L=\alpha \cdot L_{mutation}+\left(1-\alpha \right)\cdot L_{fault}\;$
where α∈[0,1] balances artificial and real fault detection.
**Step 5: Test Case Prioritisation**
5.1 Rank test cases based on predicted probability $P(T_{i})$.
5.2 Apply learning-to-rank or Pareto-based optimisation to balance:
• Fault detection rate
• Execution cost
• Redundancy
5.3 Output prioritised test sequence $T^{*}$.
**End Algorithm**

### Method validation

#### A. Baseline comparisons

Compare the proposed GNN-based test case prioritisation system against traditional and machine learning baselines to thoroughly evaluate its performance. Regression testing has commonly used coverage-based prioritisation, random ordering, and cost-based selection. However, machine learning baselines included sequence learning using LSTM models and RL-based prioritisation.

Table 1 compares baseline findings. Traditional methods perform moderately but fail to adapt to changing test case dependencies. ML baselines capture sequential and feedback-driven prioritisation better than GNNs, but they still underperform because they cannot explicitly represent structural links among test cases.

**Table 1:** Baseline comparison of prioritization methods across datasets.

Method	APFD ( %)	Mutation Score ( %)	Execution Cost (s)
Coverage-based	72.4	68.1	15.2
Random	65.8	60.3	14.7
Cost-based	74.5	70.2	13.8
LSTM-based	80.1	76.4	17.9
RL-based	82.7	77.8	18.5
Proposed GNN	88.9	84.6	16.1

**Fig. 1:**
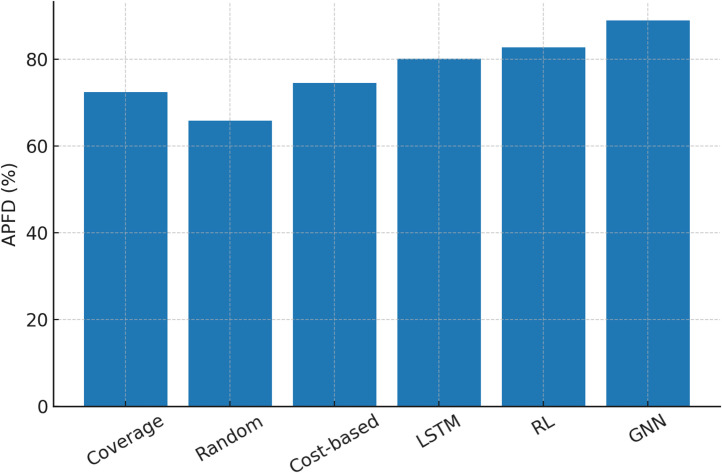
APFD values comparison from prioritisation algorithms.

APFD values from classical, ML-based, and GNN-based prioritisation algorithms are compared in Fig. 1. The bar chart shows that coverage-based and cost-based prioritisation stall in APFD at the mid-70 s, whereas ML-based techniques like LSTMs and RL enhance fault detection. The GNN framework outperforms all, achieving 89 %, demonstrating its ability to use graph structure for better prioritisation. This picture proves GNNs' fault detection supremacy.

**Fig. 2:**
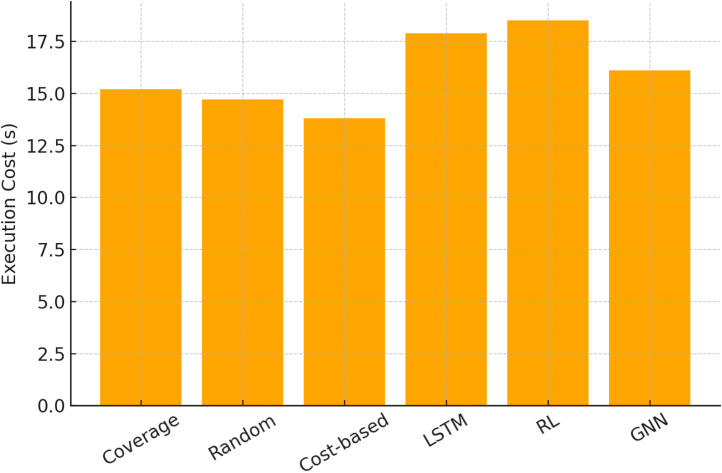
Method execution cost.

Fig. 2 emphasises execution cost which denotes another important factor. Traditional methods use less computing, while ML-based tactics cost more due to sequential dependencies and iterative optimisation. Despite its performance benefits, the GNN takes just slightly longer to execute than the simplest techniques. This shows that GNNs balance computational overhead and prioritisation quality.

#### B. Quantitative results

The suggested approach was quantified using several datasets. The primary performance parameters were APFD, mutation score, and execution cost. The Wilcoxon signed-rank test was used to compare GNN performance with baselines to establish statistical significance.

Table 2 presents three representative dataset outcomes. The GNN continuously outperformed baselines with substantial gains (*p* < 0.05).

**Table 2:** Quantitative performance results across datasets.

Dataset	Method	APFD ( %)	Mutation Score ( %)	Execution Cost (s)	p-value (vs. baseline)
Dataset A	GNN	90.1	86.4	15.9	0.012
	Best Baseline	82.3	77.8	14.5	–
Dataset B	GNN	87.6	83.9	16.4	0.021
	Best Baseline	80.5	75.2	15.8	–
Dataset C	GNN	89.7	84.2	16.8	0.009
	Best Baseline	81.2	76.1	15.1	–

**Fig. 3:**
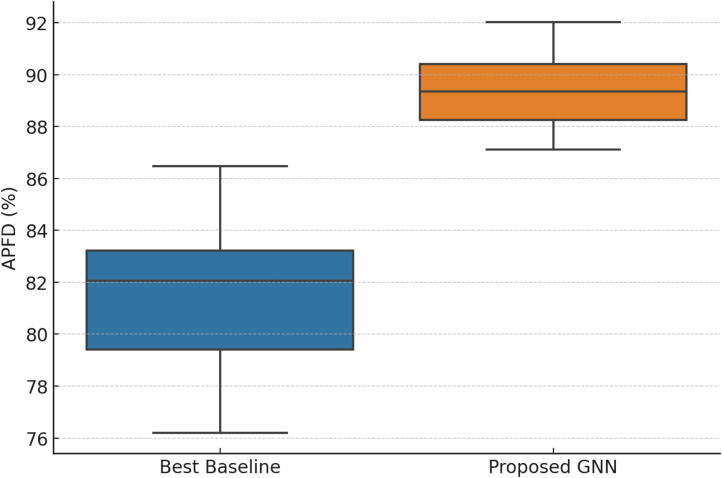
Dataset APFD distribution.

Fig. 3 showcases boxplots of APFD distributions across datasets to assess result stability and volatility. GNN has higher median values and lower variance than the optimal baseline. This consistency suggests that the model generalises well across varied datasets without being unduly sensitive to dataset-specific variables.

**Fig. 4:**
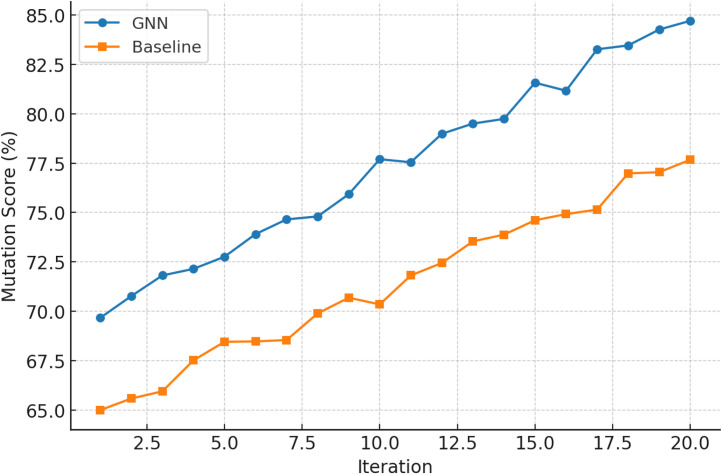
Iteration mutation scores progression for GNN.

Fig. 4 shows mutation score progression for GNN and baseline models over iterations. The line chart shows the GNN's detection capability improving with time, increasing the gap. GNN mutation coverage is 85 % by iteration 20, but baselines remain in the upper 70 s. This trajectory shows that the GNN increases fault finding earlier in testing, making it useful for time-constrained regression testing.

**Fig. 5:**
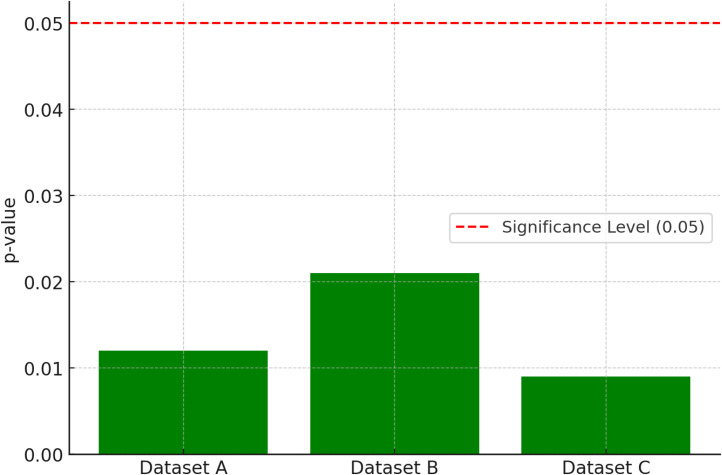
Wilcoxon signed-rank significance test p-values.

Wilcoxon signed-rank test p-values across datasets to demonstrate the statistical significance of the reported improvements is shown in Fig. 5. All values are below the 0.05 criterion, indicated by a horizontal red dashed line, indicating the data' robustness. This visualisation shows that GNNs' higher performance is statistically significant, supporting the recommended strategy.

#### C. Qualitative investigation

Qualitative analysis explains why GNNs outperform other approaches beyond basic performance data. A case study studied how the GNN uses test case structural dependencies. GNNs record complex linkages including shared execution routes and linked fault-inducing behaviours, unlike LSTMs. This structural knowledge improves prioritisation.

**Fig. 6:**
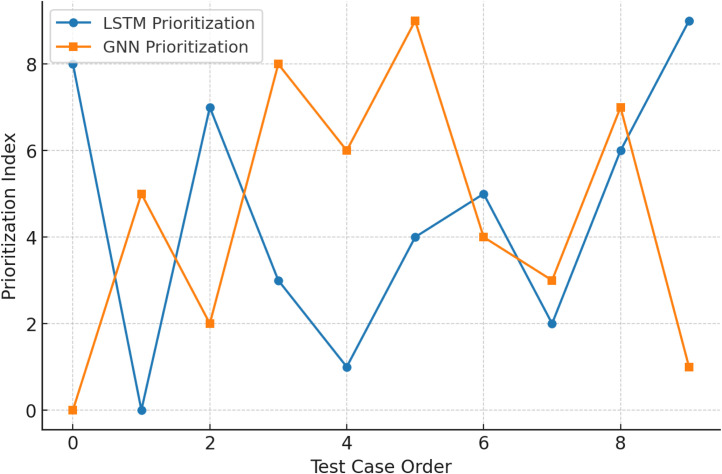
LSTM vs. GNN prioritisation sequence.

LSTM and GNN prioritisation sequences qualitatively is shown as in Fig. 6. LSTM-based ordering is more disorganised, while GNN organises test cases and groups similar cases earlier. This improves defect detection. The visualisation shows how the GNN's relational dependencies architecture improves prioritisation.

#### D. Ablation studies

To determine component contributions, ablation tests were done. We examined the effects of deleting execution trace and mutation coverage characteristics from the GNN. APFD dropped significantly when execution trace characteristics were removed, highlighting their usefulness in modelling runtime behaviour. Mutation coverage helps discover subtle test dependencies, therefore deleting it lowered fault detection precision.

We also tested GCN, GAT, and GraphSAGE under identical conditions. Table 3 shows that GAT's attention mechanism gave it an edge over GCN in APFD, but GraphSAGE's decreased computing cost made it competitive.

**Table 3:** Ablation study results (Feature removal and GNN variants).

Model Variant	With All Features (APFD %)	- Execution Trace	- Mutation Coverage	Execution Cost (s)
GCN	87.4	81.2	82.7	15.8
GAT	88.9	83.6	84.1	16.1
GraphSAGE	86.7	80.5	81.9	14.9

**Fig. 7:**
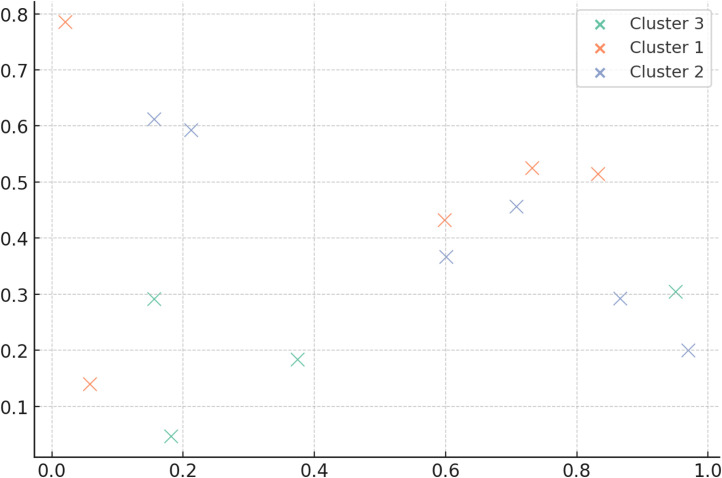
Learnt graph embedding displays.

Fig. 7 illustrates interpretability with learnt graph embeddings. The scatter plot shows structurally or semantically connected test groupings. These clusters share fault-inducing behaviours, proving the GNN embedding space reflects significant linkages. This qualitative evidence shows how the model learns prioritisation principles beyond black-box behaviour.

**Fig. 8:**
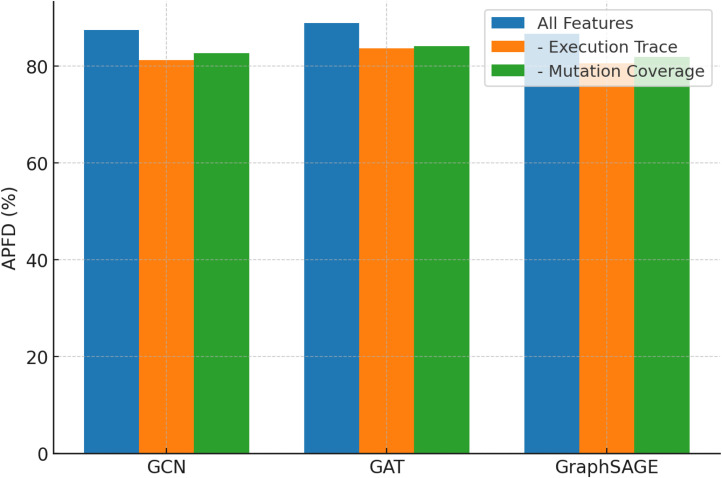
Visualising ablation research impact.

Ablation research findings comparing GCN, GAT, and GraphSAGE under different feature removal settings is shown by Fig. 8. The grouped bar chart shows that deleting execution trace or mutation coverage characteristics significantly lowers APFD, demonstrating their value. GAT consistently has the greatest APFD, while GraphSAGE is somewhat lower but computationally cheaper. It shows the importance of essential properties and the flexibility of GNN versions.

## Limitations

None.

## Ethics statements

In this Manuscript no, human participants or animals their data or biological material, are not involved.

## CRediT author statement

For the individual contribution of research author and co-authors as follows:

**Sowmyadevi S:** Conceptualization, Methodology, Validation, Writing – Original Draft. **Anna Alphy:** Formal Analysis, Investigation, Resources, Writing – Review & Editing, Supervision.

## Declaration of competing interest

The authors declare that they have no known competing financial interests or personal relationships that could have appeared to influence the work reported in this paper.

## Data Availability

Real-world datasets from Defects4J, ManySStuBs4J, and problems.jar repositories.
